# Fear of childbirth during pregnancy: associations with observed mother-infant interactions and perceived bonding

**DOI:** 10.1007/s00737-020-01098-w

**Published:** 2020-12-17

**Authors:** Fiona L. Challacombe, Selina Nath, Kylee Trevillion, Susan Pawlby, Louise M. Howard

**Affiliations:** 1 Section of Women’s Mental Health, Health Service and Population Research Department, Institute of Psychiatry, Psychology & Neuroscience, King’s College London, De Crespigny Park, SE5 8AF London, UK; 2 Division of Psychological Medicine, Institute of Psychiatry, Psychology & Neuroscience, King’s College London, London, UK

**Keywords:** Fear of childbirth, Pregnancy, Anxiety, Postpartum, Parent-child relations

## Abstract

Fear of childbirth (FOC) is a common phenomenon that can impair functioning in pregnancy but potential longer term implications for the mother-infant relationship are little understood. This study was aimed at investigating postpartum implications of FOC on the mother-infant relationship. A UK sample of 341 women in a community setting provided data on anxiety, mood and FOC in mid-pregnancy and subsequently completed self-report measures of postnatal bonding in a longitudinal cohort study. Postnatal observations of mother-infant interactions were collected and rated for a subset of 141 women. FOC was associated with maternal perception of impaired bonding, even after controlling for sociodemographic factors, concurrent depression and the presence of anxiety disorders (Coef = 0.10, 95% CI 0.07–0.14, *p* < 0.001). Observed mother-infant interactions were not associated with FOC (Coef = -0.01-0.03 CI − 0.02 to 0.02, *p* = 0.46), weakly with concurrent depression (Coef = − 0.10, CI − 0.19 to 0.00, *p* = 0.06) and not associated with anxiety disorders. The self-efficacy component of FOC was most strongly associated with lower reported bonding (Coef 0.37, 95% CI 0.25–0.49, *p* < 0.001) FOC makes a distinct contribution to perceived postpartum bonding difficulties but observed mother-infant interaction quality was not affected. This may be due to low self-efficacy impacting psychological adjustment during pregnancy. Targeted interventions during pregnancy focusing both on treatment of key childbirth fears and bonding could help women adjust earlier.

## Introduction

Pregnancy and childbirth begin a complex ongoing process of building a relationship with and parenting a child (Maas et al. 2016), but antenatal anxiety symptoms can impair some aspects of this process (Gobel et al. 2018). One form of antenatal anxiety is fear of childbirth (FOC), sometimes known as pregnancy-related anxiety or tokophobia (Nilsson et al. 2018). FOC encompasses a range of fears related to the physical welfare of both mother and child, as well as the mother’s subjective interpretations of her experiences and behaviours during birth (Nilsson et al. 2018; Sheen and Slade 2018). For some women, it causes persistent and distressing anxiety. There is mixed evidence on whether FOC is linked to requesting caesarean section (Ryding et al. 2016), but it has been associated with prolonged labour (Laursen et al. 2009) and negative health-related behaviours in pregnancy including excessive weight gain and smoking (Westerneng et al. 2017). A recent meta-analysis of international data found a prevalence of 14% for very broadly defined FOC, which may differ between cultures and settings (O’Connell et al. 2017). By contrast, we found a weighted prevalence of 3.7% using the present dataset, defined as a score of 85 or over on the most widely used measure of FOC, the Wijma Delivery Expectancy Questionnaire (W-DEQ) (Nath et al. 2019).

Severe FOC may arise from past adverse birth experiences (Stramrood and Slade 2017) or other trauma, particularly sexual abuse (Lukasse et al. 2010), but can also present as a primary phobia in the absence of such experiences (Goutaudier et al. 2018; Oliveira et al. 2017). FOC often co-occurs with other mental health problems, particularly anxiety disorders (Martini et al. 2016; Zar et al. 2002), suggesting commonalities in features or diathesis. However, it is thought to be a distinct construct (Blackmore et al. 2016).

Three studies have investigated associations between FOC and postpartum bonding and parenting. Two found a relationship between FOC and self-reported parenting stress, taking state anxiety into account (Huizink et al. 2017; Pazzagli et al. 2015), but did not adjust for concurrent depressive symptoms. A third study found that perceived antenatal bonding and concurrent depressive symptoms, but not FOC, were associated with self-reported postpartum bonding. No previous studies have used an observational measure of mother-infant interactions in addition to or instead of self-report. Observational measures capture maternal and child behaviour more objectively, the most commonly used dimension being maternal sensitivity, the ability to detect and respond appropriately to infant cues (Crittenden 2003). Maternal sensitivity in interactions is important as it can mediate the relationship between maternal psychopathology and child outcomes (Grant et al. 2010).

The current study investigated associations between antenatal FOC and postpartum bonding using both an observational measure of mother-infant interactions and self-reported bonding, adjusting for concurrent depression and anxiety disorders during pregnancy. We hypothesised that more severe FOC (assessed as a continuous variable) would be associated with poorer maternal sensitivity (using an observational measure) and impaired perceptions of bonding (using a self-reported measure) but these relationships would be attenuated by the inclusion of concurrent depression and anxiety disorders in the model.

## Methods

For the study design and population, a cohort study of 545 women were recruited antenatally in an inner-city maternity service using stratified sampling in London, UK. The WEll-being in pregNancy stuDY (WENDY) (Howard et al. 2018) was aimed at assessing the effectiveness of the commonly used ‘Whooley questions’ in identifying mental disorders in pregnant women (Whooley et al. 1997). The main study was powered to answer this question.

All women answering positively to either or both Whooley questions (W+) were invited to participate, along with a random sample of W− women who answered no to both questions. Women who declined to answer the Whooley questions, < 16 years old, had a termination or miscarriage prior to baseline interview or had booked their baby’s birth elsewhere in the UK were excluded.

Eligible women consenting to participate took part in a face-to-face interview within 3 weeks after their antenatal booking appointment (approximately 10–12-weeks’ gestation). Interviews were conducted by research midwives and postgraduate researchers trained to administer the SCID. Women were recruited between November 2014 and July 2016 from a diverse population in inner London. Language interpreters were used where needed. The study population (*n* = 545) was similar to the base population (*n* = 9963, women booking at the maternity site during the study duration) on sociodemographic factors (see Howard et al. 2018 for full details of study procedures, sampling and representativeness of the study sample).

**Fig. 1:**
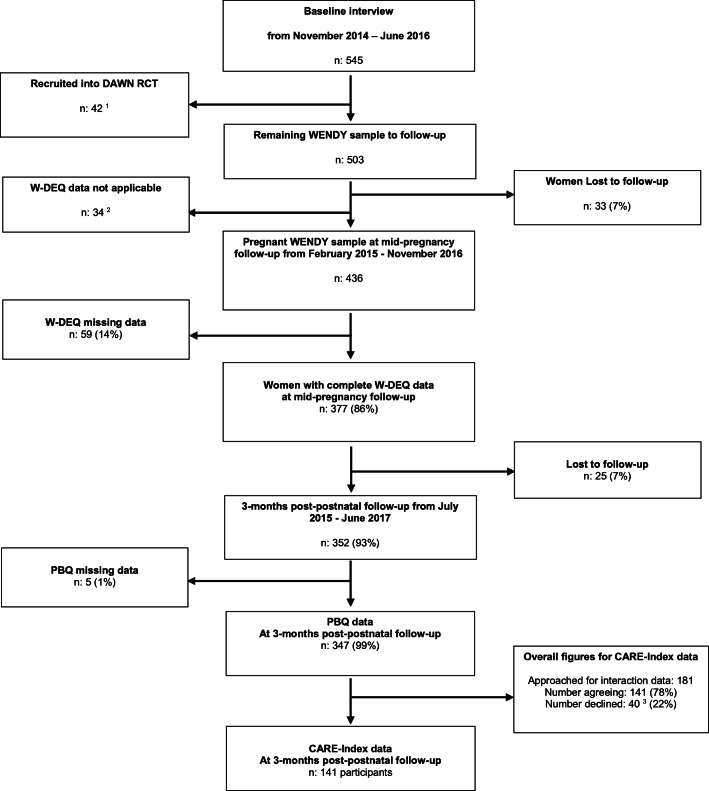
Flow chart for study population. ^1^The ESMI programme consisted of a nested randomised controlled trial (RCT) (Trevillion et al. 2016) which recruited 42 women from the WENDY study. ^2^Reasons for W-DEQ not being applicable to women: 18 (53%) had a miscarriage; 4 (12%) had a preterm birth and no longer pregnant; 12 (35%) were late bookers and were past mid-pregnancy when participating in the baseline interview. ^3^Reasons for declining mother-infant interactions: 19 (48%) uncomfortable with being recorded/videotaped; 3 (7.5%) declined home visit or any form of face-to-face visit or mother/baby not well at home visit; 3 (7.5%) baby asleep during home visit and mother did not want another home visit; 2 (5%) baby’s father did not want the baby to be recorded/videotaped; 1 (2.5%) other children upset at home visit**;** 1 (2.5%) technical problem; 11 (27%) other, e.g. woman did not want an interpreter.

Eligible women were followed up at mid-pregnancy (*n* = 436/503, 87% follow-up rate, mean pregnancy gestation 29 weeks) and approximately 3-month postpartum (*n* = 352/503, 70% follow-up rate). During the postpartum data collection period, we obtained additional funding to approach a subsample of women (*n* = 181) to participate in a home visit to collect observational mother-infant interaction data (78% agreed, *n* = 141) (see Fig. 1 for the flow chart of women through the study).

### Ethical approval

The research was approved by the National Research Ethics Service, London Committee-Camberwell St Giles (ref no 14/LO/0075). All participants provided written informed consent.

### Measures

#### Sociodemographic characteristics

Information about maternal age and education was obtained at the baseline interview. Age was treated as a continuous variable and education was divided into three categories (none/school level, college/diploma/higher certificate/vocational training and degree level/postgraduate qualification). Information regarding infant date of birth (to calculate gestational age at birth) was collected during the 3-month home visit.

#### Fear of childbirth

At mid-pregnancy (around 28 weeks’ gestation), the Wijma Delivery Expectancy/Experience Questionnaire (W-DEQ) (Wijma et al. 2002) was administered to assess FOC. This consists of 33 self-report questions designed to measure fear of labour and birth based on women’s cognitive and emotional expectations. Respondents rate each reaction in six domains on a scale of 0–5 and a single score is derived (range 0–165). Higher scores indicate greater fear, with scores of 85 or over indicating severe FOC (Calderani et al. 2019). A six-factor solution was identified by Garthus-Niegel and colleagues with fear, negative appraisal, loneliness, lack of self-efficacy, lack of positive anticipation and concerns for the child as the factors (Garthus-Niegel et al. 2011).

#### Antenatal anxiety disorders

The Structured Clinical Interview for the *Diagnostic and Statistical Manual of Mental Disorders* (4th ed.; DSM-IV) Axis I Disorders was administered (SCID, research version) (First et al. 2002) The SCID is a semi-structured diagnostic interview, to identify DSM-IV Axis I disorders. For the current analysis, the ‘anxiety disorders’ group included those who met diagnosis for one or more of generalised anxiety disorder (GAD), panic disorder, social phobia, agoraphobia, obsessive-compulsive disorder (OCD) and posttraumatic stress disorder (PTSD). Women diagnosed with specific phobia were excluded from the anxiety group for analysis, other than the one participant who had tokophobia. This was because phenomenologically, phobias are reactions to specific stimuli (such as blood, animals, environments or situations) and therefore may not be expected to be associated with disruptions in the mother-infant relationship (Kaitz et al. 2010). OCD and PTSD were included in the anxiety disorder group as the DSM-IV system was used, and there is some evidence to suggest PTSD and OCD may be associated with problems in mother-infant interactions and bonding (Challacombe et al. 2016; Ionio and Di Blasio 2014; Kaitz et al. 2010).

#### Maternal depressive symptoms

The Edinburgh Postnatal Depression Scale (EPDS) is a ten-item self-complete questionnaire for perinatal depression and validated in 20 languages (Cox et al. 1987). In a range of 0–33, scores > 12 suggest significant depression.

### Primary outcome measures

#### Mother’s perception of bonding (self-report)

The Postpartum Bonding Questionnaire (PBQ) is a 25-item validated self-report measure assessing a mother’s feeling and attitude towards her infant (Brockington et al. 2001). Items are rated on a six-point scale from 0 = ‘always’ to 5 = ‘never’; where statements reflect a negative emotion/attitude, the scoring is reversed. Total scores are generated by summing the 25 items (scores range between 0 and 125). Higher scores indicate more impaired bonding (Brockington et al. 2006).

#### CARE-index

The Child-Adult Relationship Experimental Index (CARE-Index) was used to code mother-infant interactions from recordings of a 5-min free-play session during the home visit at 3 months. The CARE-Index assesses a number of maternal (sensitive, controlling, unresponsive) and infant (cooperative, difficult, compulsive and passive) behavioural patterns. Each is rated on a scale that together totals between 0 and 14 for the mother and 0 to 14 for the infant, with higher scores indicating a higher rating of the specific pattern. This is a reliable and valid coding system for infants aged between 0 and 15 months and validated across different social classes and ethnic backgrounds (Crittenden 2003; Leventhal et al. 2004). The certified coder (with level II + research level reliability) was independent to the study team, blind to the specific aims of the study and women’s mental health status. The current analysis used maternal sensitivity, defined in the CARE-Index rating as “any pattern of behavior that pleases the infant, increases comfort and attentiveness or reduces distress and/or disengagement” (Crittenden 2003).

### Statistical analysis plan

Data management and analysis were conducted using Stata v.15. Sociodemographic characteristics of women with high (W-DEQ score of ≥ 85) and low FOC (W-DEQ < 85) were compared (age, education) using independent *t* test for continuous variables and chi-squared tests (or Fisher’s exact test for cells *n* < 5) for categorical variables. Pearson correlations were used to check inter-correlations between the PBQ and CARE-Index.

Unadjusted linear regression was run to investigate the association between maternal FOC as a continuous variable and maternal self-reported bonding with infants (PBQ score) and observed interactions (CARE-Index). Potential confounding variables were chosen a priori and based on previous literature (Stein et al. 2014) and were as follows: the presence of antenatal anxiety disorder, parity, maternal age and education and infant gestational age at birth. The multivariable regression analysis (model 2) adjusted for these variables. In a final step, we investigated whether any associations were also independent of concurrent maternal depressive symptoms. For the PBQ analysis, complete data were available for 347 women. The same analyses were repeated with maternal sensitivity as the outcome measure with complete data available on 141 women. Factors of the WDEQ were investigated for their effects on the dependent variables.

Outcome data were checked for accuracy, missing data, outliers and normality. Both primary outcome variable distributions were skewed. A sensitivity analysis was therefore conducted using log-transformed PBQ and maternal sensitivity variables which produced the same pattern of results. For ease of interpretation, the original values are presented below.

## Results

The demographics of the women for each section of the analysis are reported below.

Fourteen (3%) women had PBQ scores of > 26 (cut-off suggestive of bonding problems (Brockington et al. 2006). PBQ and maternal sensitivity scores were not correlated (− 0.03, *p* = 0.7). An initial *t* test found that there was a difference between PBQ scores for women defined as high (≥ 85) and low (< 85) on the FOC measure with a difference of 6.23 points (*p* < 0.001; 95% CI 3.04–9.05). No difference was found between FOC groups on maternal sensitivity scores, maternal age or education (Tables 1, 2, 3, 4 and 5).

**Table 1:** Sociodemographic characteristics of women contributing to PBQ and CARE-Index analysis

Characteristic	PBQ			CARE-Index		
	Low FOC	High FOC	Total	Low FOC	High FOC	Total
*n*	319	28	347	125	16	141
Maternal age mean (sd),	33.6 (5.64)	32.94 (5.23)	32.88 (5.20)	32.71 (0.47)	31.13 (1.29)	32.53 (5.27)
Education (*n*, %)						
No or school only	61(19.12)	3 (10.71)	64 (18.44)	24 (19.20)	2 (12.50)	26 (18.44)
A levels or vocational	81 (25.39)	9 (32.14)	90 (25.94)	33 (26.40)	5 (31.25)	38 (26.95)
Degree or higher	177 (55.48)	16 (57.14)	193 (55.62)	68 (54.44)	9 (56.25)	77 (54.61)
Gestational age at birth (mean, sd)	39.32 (0.08)	39.14 (0.27)	39.3 (1.48)	39.36 (0.13)	39.69 (0.30)	39.40 (1.43)
Ethnicity (*n*, %)						
White	176 (55.17)	20 (71.42)	196 (56.48)	68 (54.44)	13 (81.25)	81 (57.45)
Black	94 (29.46)	6 (21.43)	100 (28.82)	37 (29.60)	2 (12.50)	39 (27.66)
Asian	13 (4.07)	1 (3.57)	14 (4.30)	4 (3.20)	1 (6.25)	5 (3.55)
Mixed	12 (3.76)	1 (3.57)	13 (3.75)	4 (3.20)	0	4 (2.84)
Other	24 (7.52)	0	24 (6.92)	9 (7.20)	3 (18.75)	12 (8.51)
First-time parent	159 (49.84)	19 (67.85)	178 (51.30)	60 (48.0)	11 (68.75)	71 (51.06)

**Table 2:** Clinical variable mean scores—Wijma, PBQ, AD, and depression

Characteristic	PBQ			CARE-Index		
	Low FOC	High FOC	Total	Low FOC	High FOC	Total
*n*	319	28	347	125	16	141
Any antenatal anxiety disorder (*n*, %)	47 (15.06)	11 (39.28)	58 (16.71)	29 (23.58)	7 (43.75)	36 (25.50)
Panic	2(0.62)	1 (3.57)	3 (0.86)	1 (0.80)	1 (6.25)	2 (1.41)
Agoraphobia	1 (0.31)	0	1 (0.29)	1(0.80)	0	1 (0.71)
Social phobia	14 (4.38)	1 (3.57)	15 (4.32)	4 (3.20)	1 (6.25)	5 (3.55)
OCD	8 (2.50)	2 (7.14)	10 (2.88)	3 (2.40)	1 (6.25)	4 (2.84)
PTSD	6 (1.9)	1 (3.57)	7 (2.08)	4 (3.20)	1 (6.25)	5 (3.62)
GAD	23 (7.21)	7 (2.50)	31 (8.93)	17 (13.60)	4 (25.0)	21 (15.6)
Tokophobia	0	1 (3.57)	1 (0.29)	0	1 (6.25)	1 (0.71)
FOC (mean (sd))	48.45 (1.06)	94.68 (1.59)	52.18 (22.27)	49.18 (1.82)	96.38 (2.28)	54.53 (24.51)
EPDS 3 months (mean (sd))	5.91(0.27)	7.71 (0.85)	6.06 (4.84)	6.43 (0.76)	6.94 (1.07)	6.49 (5.09)
PBQ (mean (sd))	117.98 (0.41)	111.75 (0.40)	117.48 (7.78)	117.23 (0.76)	110.56 (1.93)	116.47* (8.55)
Maternal sensitivity score (mean (sd))	4.3 (0.28)	4.32 (0.79)	4.32* (2.98)	4.26 (0.27)	4.44 (0.61)	4.28 (2.98)

*139 observations in each case

**Table 3:** Correlation matrix for fear of childbirth, postnatal depressive symptoms, maternal age, gestation age, postpartum bonding and maternal sensitivity

	Fear of childbirth (WDEQ)	Depressive symptoms (EPDS)	Maternal age	Gestational age	Postpartum Bonding Questionnaire
Depressive symptoms (EPDS)	0.24***				
Maternal age	0.03	− 0.04			
Gestational age	0.02	− 0.02	− 0.02		
Postpartum Bonding Questionnaire (PBQ)	0.37***	0.33**	0.04	0.02	
Maternal sensitivity	0.01	− 0.19*	0.22*	0.05	− 0.03

*p < 0.01, **p < 0.001, and ***p < 0.0001

**Table 4:** Adjusted regression models for PBQ and maternal sensitivity

	PBQ		Maternal sensitivity	
Predictors	Coefficient (95% CI)	*p*	Coefficient (95% CI)	*p*
Fear of childbirth (WDEQ)	0.10 (0.07–0.14)	< 0.001	0.01 (− 0.01 to 0.03)	0.46
Maternal age	0.04 (− 0.11 to 0.20)	0.60	0.10 (0.00–0.21)	0.08
Infant gestational age	− 0.06 (− 0.57 to 0.45)	0.81	0.35 (− 0.01 to 0.71)	0.06
Maternal education				
No/school	Reference		Reference	
College/diploma/training	2.37 (0.08–4.66)	0.04	0.08 (− 1.47 to 1.64)	0.92
Degree and above	2.58 (0.43–4.73)	0.02	0.42 (− 1.05 to 1.89)	0.57
Concurrent depression (EPDS)	0.35 (0.19–0.51)	< 0.001	− 0.10 (− 0.20 to 0.00)	0.06
Anxiety disorder (Y/N)	− 0.12 (− 2.18 to 1.93)	0.91	− 0.69 (− 1.87 to 0.49)	0.25
First-time parent	1.47 (− 0.08 to 3.01)	0.06	− 0.76 (− 1.15 to 1.00)	0.89

**Table 5:** Adjusted regression model using WDEQ self-efficacy

	PBQ	
Predictors	Coefficient (95% CI)	*p*
Fear of childbirth-self efficacy (WDEQ)	0.37 (0.25–0.49)	< 0.001
Maternal age	0.02 (− 0.13 to 0.17)	0.82
Infant gestational age	− 0.17 (− 0.66 to 0.32)	0.50
Maternal education		
No/school	Reference	
College/diploma/training	2.37 (0.19–4.54)	0.03
Degree and above	2.33 (0.29–4.38)	0.03
Concurrent depression (EPDS)	0.41 (0.26–0.56)	< 0.001
Anxiety disorder (Y/N)	− 0.14 (− 2.10 to 1.82)	0.89
First-time parent	1.53 (− 0.44 to 3.01)	0.04

Correlations between continuous variables are presented in Table 3.

### Unadjusted univariate analysis

In the unadjusted linear regression analysis, FOC was associated with higher (impaired) scores on the PBQ (Coef = 0.13, 95% CI 0.09–0.16, *p* < 0.001). FOC was not associated with maternal sensitivity scores (Coef = -0.01-0.03 CI − 0.02 to 0.02, *p* = 0.46).

### Fear of childbirth and perceived bonding

After adjusting for maternal sociodemographic factors (age and education, parity, infant gestational age and antenatal anxiety disorder), FOC continued to be associated with higher PBQ scores, i.e. more impairment (Coef = 0.10, 95% CI 0.07–0.14, *p* < 0.001). This association remained after adjusting for the influence of concurrent depressive symptoms which were also associated (Coef = 0.35, 95% CI = 0.19–0.51, *p* < 0.001). Higher maternal education was significantly associated with higher PBQ scores (Coef = 2.37, 95% CI = 0.08–4.66, *p* = 0.04). The overall model’s adjusted *R*^2^ fit was 18.9%.

The analysis was re-run on the smaller sample of 141 who had maternal sensitivity data to check for the effect of a smaller sample size. The same pattern of results was obtained, with FOC associated with higher PBQ scores (Coef = 0.14, 95% CI 0.09–0.20, *p* < 0.001).

### Fear of childbirth and observed mother-infant interactions

The analyses were re-run using the subsample with observational data (*n* = 141), with observed maternal sensitivity as the primary outcome. FOC was not significant, nor did other variables meet criteria for significance. Marginal results associated with greater sensitivity were higher infant gestational age (Coef = 0.35; 95% CI − 0.01 to 0.71, *p* = 0.06), higher maternal age (Coef = 0.10 95% CI = 0.00–0.21, *p* = 0.08) and lower maternal depression (Coeff = − 0.10, CI − 0.19 to 0.00, p = 0.06). Maternal education was not associated with maternal sensitivity (Coef = 0.08; 95% CI − 1.47 to 1.64, *p* = 0.92). The overall model adjusted *R*^2^ fit was 8.4%.

### WDEQ factors and perceived bonding

In order to investigate whether factors of the WDEQ identified by Garthus-Niegel et al. were stronger predictors of bonding on the PBQ, they were simultaneously entered into a regression. These were fear, negative appraisals, loneliness, lack of self-efficacy, lack of positive emotions and concerns about the baby, including 25 items from the WDEQ. Only ‘lack of self-efficacy’ was significant (Coef 0.27; 95% CI 0.09–0.45, *p* = 0.003), with the adjusted *R*^2^ fit being 14.0%. Using this factor in the overall model, the pattern of results remained similar, with lack of self-efficacy (Coef 0.379, 95% CI 0.26–0.50, *p* < 0.001), depression (Coef 0.41, 95% CI 0.26–0.56, *p* < 0.001) and education (Coef 2.37, 95% CI 0.19–4.54, *p* = 0.03) significant, and a similar amount of variance explained by the overall model (adjusted *R*^2^ = 19.4). However, being a first-time mother was also associated (Coef = 1.53, 95% CI − 0.04 to 3.01, *p* = 0.04).

## Discussion

We found that fear of childbirth (FOC) during pregnancy was associated with more impaired perceived bonding at 3-month postpartum but not with lower sensitivity in observed mother-infant interactions. The association between FOC and perceived bonding difficulties remained even after adjusting for concurrent postpartum depressive symptoms and the presence of antenatal anxiety disorder, suggesting a relatively robust effect.

Consistent with our results, FOC has been related in previous studies to lower self-reported postnatal bonding (Huizink et al. 2017) and parental stress (Pazzagli et al. 2015). However, this study is the first to our knowledge to find that FOC is not associated with lower maternal sensitivity on an observational measure. These combined findings suggest that perceived and observed parenting may be different concepts to assess when considering the impact of parental psychopathology. Further to this, the measures did not correlate. Sensitivity has a moderate relationship with attachment in meta-analyses (De Wolff and van Ijzendoorn 1997) but the relationship with perceived bonding may be weaker or may change over time as attachment is established. A low number of women reported impaired bonding over a clinical threshold in this sample; therefore, there may not have been enough power to accurately detect a relationship with sensitivity.

Contrary to hypothesis, antenatal anxiety disorders were not associated with perceived or observed bonding when included in the model with FOC. Previous studies of mothers with generalised anxiety disorder and obsessive-compulsive disorder found some interference with postpartum interactions (Challacombe et al. 2016; Stein et al. 2012). FOC is therefore an independent anxiety-related risk factor for early adjustment to parenthood in terms of perceived bonding (Gobel et al. 2018). Preoccupation with pregnancy fears at the expense of gradual adjustment to the process of becoming a parent is one possible mechanism (Dubber et al. 2015). The concept of FOC has several components including fears relating to the infant as well as fears specifically around childbirth (Bayrampour et al. 2016). Our results highlighted that lack of self-efficacy in the birth experience was most strongly related to perceived bonding, rather than fear of specific outcomes. Both low efficacy regarding birth and bonding may be related to a general unconfident thinking style and negative self-appraisal, which could be identified and addressed early in pregnancy (Schmidt et al. 2017). FOC was related to depressive symptoms and the concept may therefore include elements related to both anxiety and depression.

Future studies could investigate whether a traumatic birth in which fears were realised mediates the relationship between FOC and postnatal bonding and whether problems persist. Perceived difficulties in bonding measured by the PBQ have been associated with parental stress and negative child outcomes (de Cock et al. 2017; Fuchs et al. 2016). Perceived bonding is therefore of likely clinical importance, and the PBQ could be a useful way to identify dyads at risk of future issues.

There is considerable evidence of a relationship between maternal depressive symptoms and difficulties both in perceived bonding and interactions (Field 2010). Low mood was strongly associated with perceived bonding and weakly associated with observed interactions in the current study. Depression remains an important factor to be carefully assessed and addressed.

This study highlights that fear of childbirth is important to assess for all pregnant women, not only because it can have bearings on the experience of pregnancy and birth and antenatal anxiety symptoms but also because it could affect postnatal bonding. The WDEQ is long; brief, valid and clinically useful measures for FOC need further development, and a focus on self-efficacy in pregnancy and birth may be particularly useful. Detailed investigation of the particular strategies used by women to cope with FOC such as behavioural and emotional avoidance and the impact on antenatal attachment representations could inform understanding of mechanisms and the development of effective interventions. The individual aetiology of FOC is likely to be particularly important in understanding its postnatal significance, particularly in any relationship with previous antenatal loss, traumatic birth or childhood trauma (Robertson Blackmore et al. 2011). There is currently little evidence on which to base interventions although effective treatment of antenatal anxiety is a research and clinical priority. Further research is needed on whether effective antenatal treatments for FOC can ameliorate anxiety and low mood and potentially impact infant temperament or mother-infant bonding postnatally (Rouhe et al. 2015; Netsi et al. 2015).

Strengths of the study were the use of observed mother-infant interactions rather than exclusively self-report measures, a large sample size and use of a diagnostic interview to establish diagnoses. Limitations include the lack of a maternal-fetal attachment measure to assess bonding longitudinally or a continuous anxiety measure postnatally to assess the impact of concurrent anxiety symptoms on functioning. Although it was not possible to reassess anxiety disorders postnatally, these were assessed robustly in the antenatal period and some continuity is likely (Martini et al. 2015). The postpartum play task might not have captured difficulties in interactions if the mother was not feeling anxious at the time (Stein et al. 2012). However, a postnatal task related to FOC would be difficult to devise; our findings are consistent with there being no global effect of FOC on maternal sensitivity.

In summary, there are evident but subtle impacts of FOC for women in the postpartum. Antenatal interventions for FOC that encompass self-efficacy and the possible impact on the mother-infant relationship could potentially prevent postpartum difficulties.

## Data Availability

Not available due to small no. of some cells and potential identifiability.

## Abbreviations

FOC: Fear of childbirth

## References

[CR1] Bayrampour H, Ali E, McNeil DA, Benzies K, MacQueen G, Tough S (2016). Pregnancy-related anxiety: a concept analysis. Int J Nurs Stud.

[CR2] Blackmore ER, Gustafsson H, Gilchrist M, Wyman C, G O’Connor T (2016). Pregnancy-related anxiety: evidence of distinct clinical significance from a prospective longitudinal study. J Affect Disord.

[CR3] Brockington IF, Oates J, George S, Turner D, Vostanis P, Sullivan M, Loh C, Murdoch C (2001). A screening questionnaire for mother-infant bonding disorders. Arch Women’s Mental Health.

[CR4] Brockington IF, Fraser C, Wilson D (2006). The Postpartum Bonding Questionnaire: a validation. Arch Women’s Mental Health.

[CR5] Calderani E, Giardinelli L, Scannerini S, Arcabasso S, Compagno E, Petraglia F, Ricca V (2019). Tocophobia in the DSM-5 era: outcomes of a new cut-off analysis of the Wijma delivery expectancy/experience questionnaire based on clinical presentation. J Psychosom Res.

[CR6] Challacombe FL, Salkovskis PM, Woolgar M, Wilkinson EL, Read J, Acheson R (2016). Parenting and mother-infant interactions in the context of maternal postpartum obsessive-compulsive disorder: effects of obsessional symptoms and mood. Infant Behav Dev.

[CR7] Cox JL, Holden JM, Sagovsky R (1987). Detection of postnatal depression - development of the 10-item Edinburgh Postnatal Depression Scale. Br J Psychiatry.

[CR8] Crittenden PM (2003) CARE index manual. Family Relations Institute, Miami

[CR9] de Cock ESA, Henrichs J, Klimstra TA, Janneke B. M. Maas A, Vreeswijk CMJM, Meeus WHJ, van Bakel HJA (2017) Longitudinal associations between parental bonding, parenting stress, and executive functioning in toddlerhood. 26:1723–1733. 10.1007/s10826-017-0679-7 10.1007/s10826-017-0679-7 PMC5429904 28572718

[CR10] De Wolff M, van Ijzendoorn MH (1997). Sensitivity and attachment: a meta-analysis on parental antecedents of infant attachment. Child Dev.

[CR11] Dubber S, Reck C, Muller M, Gawlik S (2015). Postpartum bonding: the role of perinatal depression, anxiety and maternal-fetal bonding during pregnancy. Arch Women’s Mental Health.

[CR12] Field T (2010). Postpartum depression effects on early interactions, parenting, and safety practices: a review. Infant Behav Dev.

[CR13] First MB, Spitzer RL, Gibbon M, Williams JB (2002) Structured clinical interview for DSM-IV-TR axis I disorders, research version, patient edition. SCID-I/P

[CR14] Fuchs A, Mohler E, Reck C, Resch F, Kaess M (2016). The early mother-to-child bond and its unique prospective contribution to child behavior evaluated by mothers and teachers. Psychopathology.

[CR15] Garthus-Niegel S, Størksen HT, Torgersen L, Von Soest T, Eberhard-Gran M (2011). The Wijma Delivery Expectancy/Experience Questionnaire – a factor analytic study. J Psychosom Obstet Gynecol.

[CR16] Gobel A, Stuhrmann LY, Harder S, Schulte-Markwort M, Mudra S (2018). The association between maternal-fetal bonding and prenatal anxiety: an explanatory analysis and systematic review. J Affect Disord.

[CR17] Goutaudier N, Bertoli C, Sejourne N, Chabrol H (2018) Childbirth as a forthcoming traumatic event: Pretraumatic stress disorder during pregnancy and its psychological correlates. J Reprod Infant Psychol. 10.1080/02646838.2018.1504284 10.1080/02646838.2018.1504284 30095279

[CR18] Grant K-A, McMahon C, Reilly N, Austin M-P (2010). Maternal sensitivity moderates the impact of prenatal anxiety disorder on infant mental development. Early Hum Dev.

[CR19] Howard LM (2018). Accuracy of the Whooley questions and the Edinburgh Postnatal Depression Scale in identifying depression and other mental disorders in early pregnancy. Br J Psychiatry.

[CR20] Huizink A, Menting B, Moor M, Verhage M, Kunseler F, Schuengel C, Oosterman M (2017) From prenatal anxiety to parenting stress: a longitudinal study. Arch Women’s Mental Health. 10.1007/s00737-017-0746-5 10.1007/s00737-017-0746-5 PMC5599437 28634716

[CR21] Ionio C, Di Blasio P (2014). Post-traumatic stress symptoms after childbirth and early mother–child interactions: an exploratory study. J Reprod Infant Psychol.

[CR22] Kaitz M, Maytal HR, Devor N, Bergman L, Mankuta D (2010). Maternal anxiety, mother–infant interactions, and infants’ response to challenge. Infant Behav Dev.

[CR23] Laursen M, Johansen C, Hedegaard M (2009). Fear of childbirth and risk for birth complications in nulliparous women in the Danish National Birth Cohort BJOG : an. Int J Obstet Gynaecol.

[CR24] Leventhal A, Jacobsen T, Miller L, Quintana E (2004) Caregiving attitudes and at-risk maternal behavior among mothers with major mental illness. Psychiatr Serv:55 10.1176/appi.ps.55.12.1431 15572573

[CR25] Lukasse M, Vangen S, Oian P, Kumle M, Ryding EL, Schei B (2010). Childhood abuse and fear of childbirth-a population-based study. Birth.

[CR26] Maas AJBM, de Cock ESA, Vreeswijk CMJM, Vingerhoets AJJM, van Bakel HJA (2016). A longitudinal study on the maternal–fetal relationship and postnatal maternal sensitivity. J Reprod Infant Psychol.

[CR27] Martini J, Petzoldt J, Einsle F, Beesdo-Baum K, Hofler M, Wittchen HU (2015). Risk factors and course patterns of anxiety and depressive disorders during pregnancy and after delivery: a prospective-longitudinal study. J Affect Disord.

[CR28] Martini J, Asselmann E, Einsle F, Strehle J, Wittchen HU (2016). A prospective-longitudinal study on the association of anxiety disorders prior to pregnancy and pregnancy- and child-related fears. J Anxiety Disord.

[CR29] Nath S, Busuulwa P, Ryan EG, Challacombe FL, Howard LM (2019) The characteristics and prevalence of phobias in pregnancy. Midwifery. 10.1016/j.midw.2019.102590 10.1016/j.midw.2019.102590 31864080

[CR30] Netsi E, Evans J, Wulff K, O’Mahen H, Ramchandani PG (2015). Infant outcomes following treatment of antenatal depression: findings from a pilot randomized controlled trial. J Affect Disord.

[CR31] Nilsson C (2018). Definitions, measurements and prevalence of fear of childbirth: a systematic review. BMC Pregnancy Childbirth.

[CR32] O’Connell MA, Leahy-Warren P, Khashan AS, Kenny LC, O’Neill SM (2017). Worldwide prevalence of tocophobia in pregnant women: systematic review and meta-analysis. Acta Obstet Gynecol Scand.

[CR33] Oliveira AGS, Reichenheim ME, Moraes CL, Howard LM, Lobato G (2017). Childhood sexual abuse, intimate partner violence during pregnancy, and posttraumatic stress symptoms following childbirth: a path analysis. Arch Women’s Mental Health.

[CR34] Pazzagli C, Laghezza L, Capurso M, Sommella C, Lelli F, Mazzeschi C (2015). Antecedents and consequences of fear of childbirth in nulliparous and parous women. Infant Ment Health J.

[CR35] Robertson Blackmore E, Cote-Arsenault D, Tang W, Glover V, Evans J, Golding J, O’Connor TG (2011). Previous prenatal loss as a predictor of perinatal depression and anxiety. Br J Psychiatry.

[CR36] Rouhe H, Salmela-Aro K, Toivanen R, Tokola M, Halmesmaki E, Ryding E-L, Saisto T (2015). Group psychoeducation with relaxation for severe fear of childbirth improves maternal adjustment and childbirth experience-a randomised controlled trial. J Psychosom Obstet Gynecol.

[CR37] Ryding EL, Lukasse M, Kristjansdottir H, Steingrimsdottir T, Schei B (2016). Pregnant women’s preference for cesarean section and subsequent mode of birth-a six-country cohort study. J Psychosom Obstet Gynecol.

[CR38] Schmidt D, Seehagen S, Hirschfeld G, Vocks S, Schneider S, Teismann T (2017). Repetitive negative thinking and impaired mother-infant bonding: a longitudinal study. Cogn Ther Res.

[CR39] Sheen K, Slade P (2018). Examining the content and moderators of women’s fears for giving birth: a meta-synthesis. J Clin Nurs.

[CR40] Stein A, Craske MG, Lehtonen A, Harvey A, Savage-McGlynn E, Davies B, Goodwin J, Murray L, Cortina-Borja M, Counsell N (2012). Maternal cognitions and mother-infant interaction in postnatal depression and generalized anxiety disorder. J Abnorm Psychol.

[CR41] Stein A, Pearson RM, Goodman SH, Rapa E, Rahman A, McCallum M, Howard LM, Pariante CM (2014). Effects of perinatal mental disorders on the fetus and child. Lancet.

[CR42] Stramrood C, Slade P (2017). A woman afraid of becoming pregnant again: posttraumatic stress disorder following childbirth. Bio-psycho-social obstetrics and gynecology: a competency-oriented approach.

[CR43] Westerneng M, Witteveen AB, Warmelink J, Spelten E, Honig A, de Cock P (2017). Pregnancy-specific anxiety and its association with background characteristics and health-related behaviors in a low-risk population. Compr Psychiatry.

[CR44] Whooley MA, Avins AL, Miranda J, Browner WS (1997). Case-finding instruments for depression. Two questions are as good as many. J Gen Intern Med.

[CR45] Wijma K, Alehagen S, Wijma B (2002). Development of the Delivery Fear Scale. J Psychosom Obstet Gynecol.

[CR46] Zar M, Wijma K, Wijma B (2002). Relations between anxiety disorders and fear of childbirth during late pregnancy. Clin Psychol Psychother.

[CR47] Trevillion K, Ryan EG, Pickles A, Heslin M, Byford S, Nath S, Bick D, Milgrom J, Mycroft R, Domoney J, Pariante C, Hunter MS, Howard LM (2020). An exploratory parallel-group randomised controlled trial of antenatal Guided Self-Help (plus usual care) versus usual care alone for pregnant women with depression: DAWN trial. Journal of Affective Disorders.

[CR48] Trevillion K, Domoney J, Pickles A, Bick D, Byford S, Heslin M, Milgrom J, Mycroft R, Pariante C, Ryan E, Hunter M, Howard LM (2016). Depression: an exploratory parallel-group randomised controlled trial of Antenatal guided self help for WomeN (DAWN): study protocol for a randomised controlled trial. Trials.

